# Screening of native microalgae species for carbon fixation at the vicinity of Malaysian coal-fired power plant

**DOI:** 10.1038/s41598-020-79316-9

**Published:** 2020-12-18

**Authors:** Liyana Yahya, Razif Harun, Luqman Chuah Abdullah

**Affiliations:** 1 Department of Chemical and Environmental Engineering, Faculty of Engineering, Universiti Putra Malaysia, 43400 Serdang, Selangor Malaysia; 2 Centre of Bioenergy and Sustainability, Renewable Energy and Green Technology, TNB Research Sdn. Bhd., No. 1, Lorong Ayer Itam, Kawasan Institusi Penyelidikan, 43000 Kajang, Selangor Malaysia

**Keywords:** Environmental impact, Environmental biotechnology, Archaea

## Abstract

Global warming has become a serious issue nowadays as the trend of CO_2_ emission is increasing by years. In Malaysia, the electricity and energy sector contributed a significant amount to the nation’s CO_2_ emission due to fossil fuel use. Many research works have been carried out to mitigate this issue, including carbon capture and utilization (CCUS) technology and biological carbon fixation by microalgae. This study makes a preliminary effort to screen native microalgae species in the Malaysian coal-fired power plant’s surrounding towards carbon fixation ability. Three dominant species, including *Nannochloropsis* sp., *Tetraselmis* sp., and *Isochrysis* sp. were identified and tested in the laboratory under ambient and pure CO_2_ condition to assess their growth and CO_2_ fixation ability. The results indicate *Isochrysis* sp. as the superior carbon fixer against other species. In continuation, the optimization study using Response Surface Methodology (RSM) was carried out to optimize the operating conditions of *Isochrysis* sp. using a customized lab-scale photobioreactor under simulated flue gas exposure. This species was further acclimatized and tested under actual flue gas generated by the power plant. *Isochrysis* sp. had shown its capability as a carbon fixer with CO_2_ fixation rate of 0.35 gCO_2_/L day under actual coal-fired flue gas exposure after cycles of acclimatization phase. This work is the first to demonstrate indigenous microalgae species' ability as a carbon fixer under Malaysian coal-fired flue gas exposure. Thus, the findings shall be useful in exploring the microalgae potential as a biological agent for carbon emission mitigation from power plants more sustainably.

## Introduction

Over the past few years, average global temperature has increased significantly due to the increment of greenhouse gas emission and this trend is accelerating. According to the data reported by National Oceanic and Atmospheric Administration (NOAA), carbon dioxide (CO_2_) concentration in the atmosphere is increasing and has reached an average of 400 parts per million (ppm) in 2019 whereby the safe level is only at 350 ppm. These have a significant impact on global warming and ocean acidification^1^. Referring to the BP Statistical Review of World Energy 2019, growth in energy demand is one of the reasons for a 2% increment of carbon dioxide (CO_2_) emissions from the energy industry, which is equivalent to 250.3 million tonnes^2^. The United Nations Framework Convention on Climate Change’s (UNFCCC) 21st Conference of Parties (COP21) has become a critical turning point for the global community where Malaysia had committed to reduce CO_2_ emission per unit of GDP by 45% in 2030. Being one of the largest CO_2_ emitters, the energy industry could contribute in achieving this national target. A transition towards renewable energy and alternative fuels is among proactive approaches in reducing carbon dioxide emission; however, the means in dealing with carbon dioxide itself through capturing or sequestration is an alternative way to mitigate this issue. On the other hand, few carbon capture technologies are available with different mechanisms and maturity levels, including pre-, during, and post-combustion approaches^3^.

The CO_2_ sequestration by microalgae is considered to be a sustainable alternative approach as it can sequester CO_2_ naturally into O_2_ and organic matter through the photosynthesis process^4–6^. In terms of biomass, this organic matter with suitable pre-treatment processes can be converted into valuable downstream products, including biofuel, nutritional, aquaculture food, fertilizers and etc.^7,8^. Intensive researches have been carried out to develop a feasible and efficient system for CO_2_ mitigation in industrial scale^9–12^. However, the efficiency of this biological CO_2_ sequestration depends on certain parameters including identification of suitable algae strain, photobioreactor design, pH, source of CO_2_ supply, temperature and nutrient media^13^. Various studies have been reported on algae capabilities to grow under different flue gas exposure. For example, single phototroph species such as *Tetraselmis* sp. and *Chlorella* sp. were reported to grow well when exposed to flue gas composition containing 10–15% of CO_2_ concentration^5,8,14^. Also, some studies showed the promising results by using consortia species, for example, mixed freshwater culture with *Desmodesmus* sp. as the dominant species were cultured under actual flue gas that contains up to 11% of CO_2_^4^ and *Spirulina platensis* with mixed algal culture were fed with flue gas at CO_2_ concentration up to 15% v/v^15^.

Most algae research in Malaysia focuses on the downstream application at a laboratory scale whereby to achieve economic viability and sustainability of this technology, the challenges in both upstream and downstream processes need to be appropriately addressed. As there are little works on the upstream process, this study explores the potential of native microalgae species as the biological carbon fixers under the Malaysian coal-fired flue gas exposure. The significance of utilizing native microalgae species instead of common species is to expedite the acclimatization period and ease-out the in-situ biological CO_2_ fixation process due to the robust and conducive environment for optimum growth of the species. The optimized native microalgae species obtained at laboratory conditions were then tested under actual coal-fired flue gas to screen their potential in mitigating CO_2_ emission from industries. A central composite design (CCD) was employed to determine the effect of four operating parameters including gas flow rate, temperature, luminance and pH to obtain the maximum carbon fixation rate ability of the microalgae. The work is significantly important to demonstrate the potential of native microalgae species as the biological carbon fixers towards a more circular economy and environmentally sustainable coal-fired power stations in the long term.

## Materials and methods

### Sample collection

**Figure 1:**
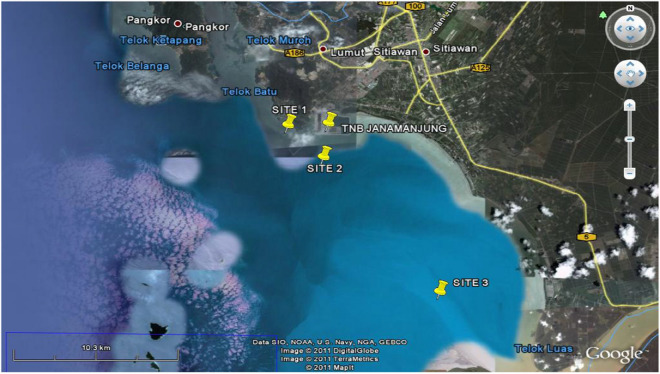
Spatial map of sampling sites (figure was taken using google satellite map and marked based on sampling site, https://satellite-map.gosur.com).

The sampling of native microalgae species was conducted at the Sultan Azlan Shah TNB Power Station, Perak, Malaysia. This coal-fired power plant generates 3100 MW of electricity and located on a 325 hectare wholly man-made island off the Lekir coast in Janamanjung, Perak, Malaysia. Three sampling locations were identified in the vicinity based on the different site characteristics, as tabulated in Table 1 and mapped in Fig. 1.

**Table 1:** Details of the sampling sites.

Site ID	GPS coordinate	Approximate distance from power plant	Luminance (klux)	Temperature (°C)	pH	Dissolved O_2_ (%)	Phosphate (mg/L)	Remarks
Site 1	N 4° 9.435′; E 100° 37.079′	2.5 km	26.6 ± 2.0	30.5 ± 0.3	7.97 ± 0.05	89.85 ± 0.5	2.05 ± 0.5	Near lighthouse at Teluk Rubiah
Site 2	N 4° 8.195′; E 100° 38.103′	2.4 km	31.25 ± 3.0	29.6 ± 0.3	8.36 ± 0.3	92.3 ± 0.3	2.50 ± 0.5	Near coal jetty
Site 3	N 4° 2.675′; E 100° 41.859′	14 km	8.05 ± 2.0	29.0 ± 0.4	7.50 ± 0.5	83.3 ± 0.5	> 4	At mouth of Perak river

**Figure 2:**
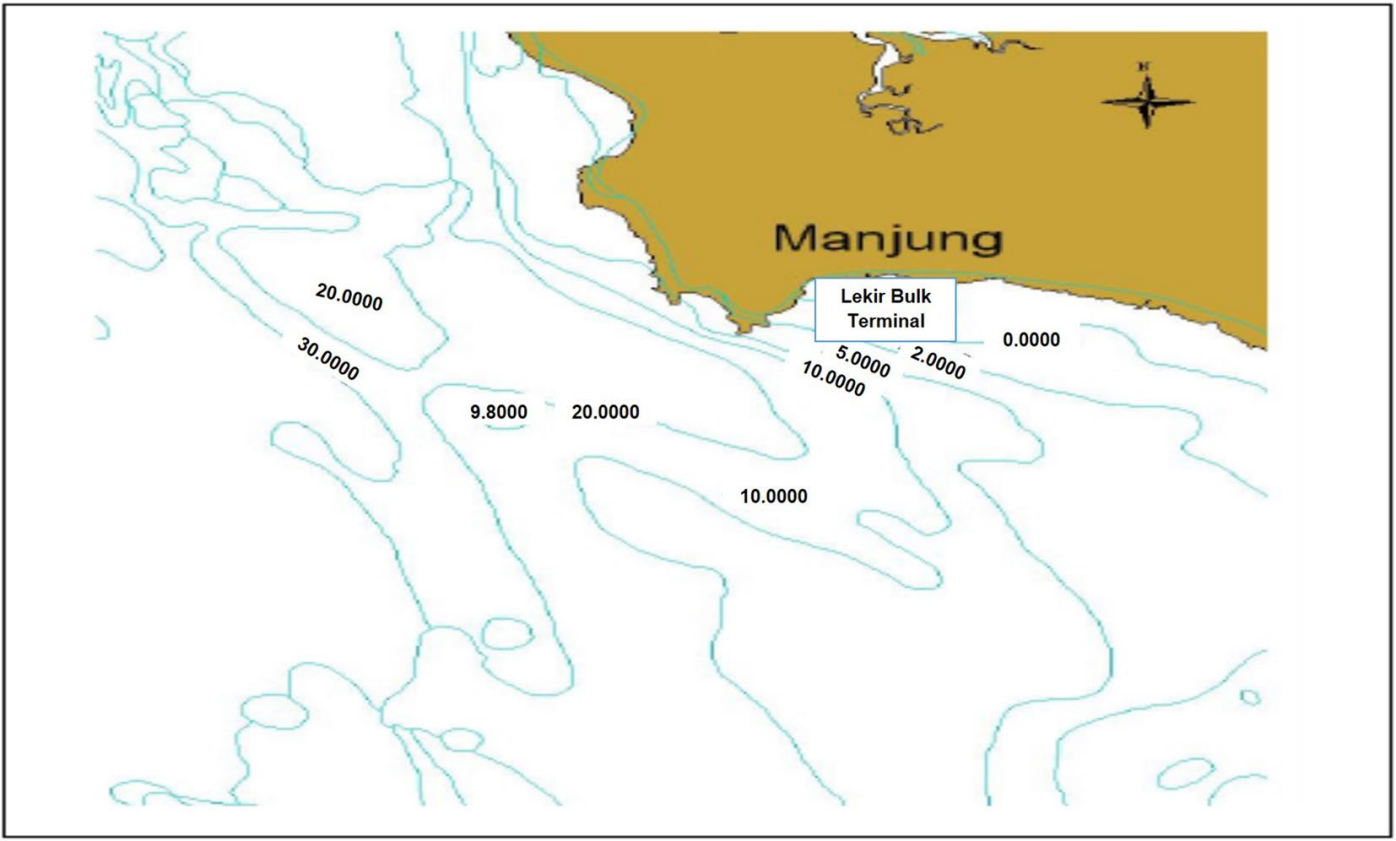
Bathymetry chart, 5 km from Janamanjung power station.

The GPS coordinate was measured using Garmin GPSMAP Handheld Navigation Device. Site #1 has less human activities in the area where it is located within a bay of Teluk Rubiah where a resort was once operated. The site #2 is located at the seawater discharge point of the station and site #3 has rich samples of microalgae derived from rich river discharges. All the sites’ depth was also checked to be at least 10 m deep as shown by a bathymetry chart around the power station in Fig. 2.

The samples were collected using a dip net method where a plankton net of 35 μm mesh size, 25 cm mouth diameter and 1.5 m long were used. The net was submerged at least 1.5 m below the surface of water and then pulled it up vertically using a rope and pulley assembly. The net was later sprayed with in-situ sea water before the liquid was collected by a sampling bottle attached at the end of the net. The physical properties like luminance, temperature, pH and dissolved O_2_ were measured at the site using lux meter and Eutech CyberScan PCD 650 portable multi meter. Samples were kept in 500 ml plastic bottles, labeled and deposited in a cool-box during transportation to laboratory and the phosphate content (PO_4_^3−^) was later determined using HACH standard procedure in the laboratory. The physical characteristics of the sampling at different sites are shown in Table 1.

### Isolation of microalgae

The collected sample was first enriched with Conway media as the broad spectrum medium right after collection to allow the entire algae population to flourish. The growing culture was then introduced to a tolerable level of antibiotics with penicillin levels ranging from 20–500 mg/l to eliminate contaminants. Air was bubbled through the culture with continuous light supply. After 3–4 days, a narrow range spectrum media was introduced to provide conducive environment for the dominant species to survive. Small volumes (15 ml) samples from the enriched cultures were centrifuged at 3000 rpm for 15 min. The supernatant was removed, and cells were re-suspended in fresh medium. The centrifugation process was repeated for few times to expel the most of microorganisms presented in algal sample. The cells were then streaked onto agar plates using aseptic technique and kept for at least seven days to grow the microalgae. Repeated streak-plating was carried out to peak up a single colony from earlier streaked plates. The single colonies were picked up by a loop and allowed to grow in tubes and vial. The colony was examined for its purity by checking the cells under microscope. Identification of species was done by visual inspection of the morphologies observed under a microscope with reference to the Algae Identification Field Guide and online database^16–18^.

### Laboratory microalgal cultivation

The laboratory microalgal cultivation was perform at the Microbiology Laboratory, TNB Research Sdn. Bhd., Kajang, Selangor. The isolated microalgae species were cultured in 2L flask using a f/2medium composed of NaCl (24.32 g/l), MgCl_2_ (5.14 g/l), CaCl_2_ (1.14 g/l), KCl (0.69 g/l), NaHCO_3_ (0.2 g/l), KBr (0.1 g/l), H_3_BO_3_ (0.027 g/l), SrCl_2_ (0.026 g/l), NH_4_Cl (0.0064 g/l), NaF (0.003 g/l), NaSiO_3_ (0.002 g/l), FePO_4_ (0.001 g/l), NaNO_3_ (75 g/l), NaH_2_PO_4_ (5 g/l), Na_2_EDTA (4.36 g/l), FeCl_3_.6H_2_O (3.15 g/l), trace metal stock solution (1.0 ml/l) and vitamin stock solution (0.5 ml/l). The medium preparation was performed in a biohazard laminar flow to minimize contamination. The microalgae were cultivated for maximum of 14 days at room temperature with average of 26 °C and illuminated with 18 W fluorescent bulb for 12 h with an average luminosity of 800 lx using timer switch. Air pump with 0.045 MPa compression and 150 L/min maximum capacity was used and to supply aeration for the algae culture. The samples were taken daily to monitor their growth performance in terms of cell density, chlorophyll A and phaeophytin content.

### Carbon fixation experiment

**Figure 3:**
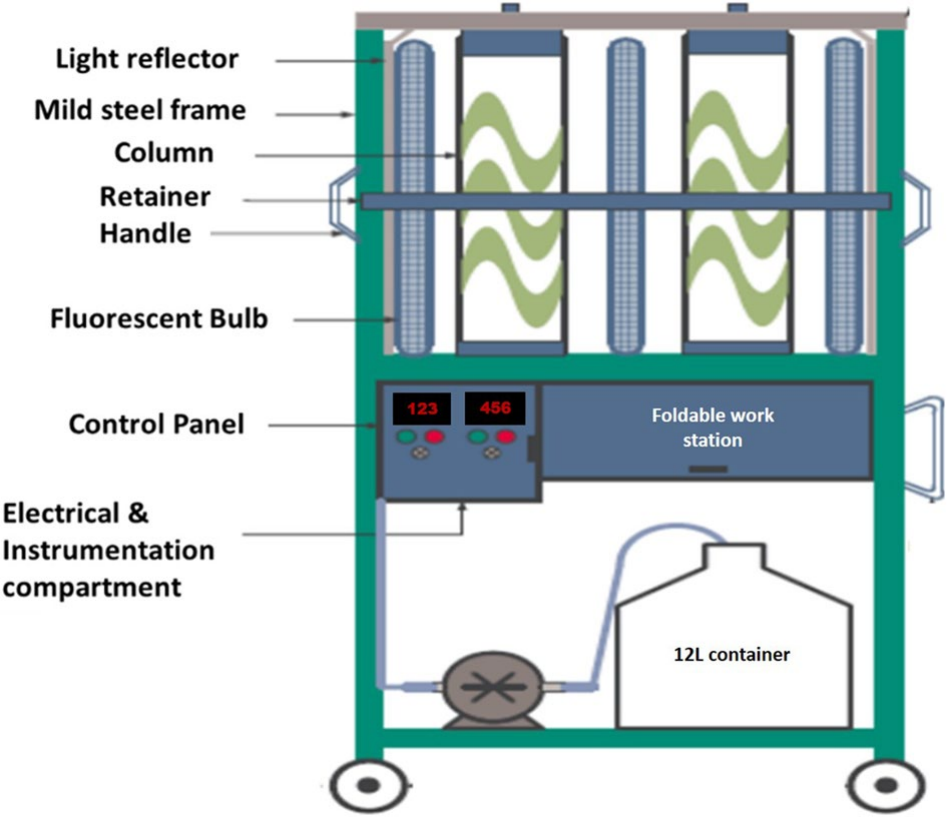
Layout of customize lab-scale photobioreactor.

A custom-made two units of bubbling laboratory scale photobioreactor (PBR) was used with a capacity of 10L each. The reactor was made from polycarbonate due to its high resistance and transparency of 92%^19^. The PBR temperature can vary from − 10 to 100 °C with the help of chiller/heater. The system was equipped with other instruments including pH sensor, dissolved O_2_ sensor, thermocouple, fluorescent bulbs with timer-controller and data acquisition system for automatic data logging. The layout of the PBR is shown in Fig. 3. The simulated flue gas was supplied throughout the cultivation period and its composition is listed in Table 2. The microalgae were cultivated for up to 14 days and the operating conditions were set based on the optimization statistical model.

**Table 2:** Simulated flue gas composition.

Gas compound	Composition
Carbon dioxide, CO_2_	4%
Oxygen, O_2_	3%
Carbon monoxide, CO	105 mg/m^3^
Nitrogen dioxide, NO_2_	272 mg/m^3^
Sulphur dioxide, SO_2_	121 mg/m^3^
Nitrogen, N_2_	Balance

The calculation of carbon fixation as shown in Eq. 1 was adopted from balanced photosynthesis formula on the ratio between CO_2_ moles and molecular formula of biomass which is about 1.8 g of CO_2_ can be fixed by 1 g of microalgae^20^:

1 $$4{\text{CO}}_{2} + {\text{nutrient}} + {\text{H}}_{2} {\text{O}} + {\text{light}} \to 4{\text{CO}}_{0.48} {\text{H}}_{1.83} {\text{N}}_{0.11} {\text{P}}_{0.01} + 3\left( {1/2} \right){\text{O}}_{2}$$

The doubling time of the microalgae cells is the time taken for the population to double its growth and was derived from Monod equation, as in Eq. 2^20^:

2 $${\text{T}}_{{\text{d}}} = \left( {{\text{t}}_{2} - {\text{t}}_{1} } \right) \times \frac{\ln (2)}{{\ln \left( {{\text{N}}_{{\text{t}}} } \right) - \ln \left( {{\text{N}}_{{\text{o}}} } \right)}}$$

where T_d_—doubling time (time taken for population to double) (day); t_2_—last day of the population growth curve (day); t_1_—first day of the population growth curve (day); N_t_—number of cells on the last cultivation period; N_o_—number of cells on the first cultivation period.

### Analysis of parameters monitoring

#### Cell count measurement

Cell counting was performed with a Neubauer improved haemacytometer set from Hirschmann Laborgerate. One drop of microalgae sample was transferred to the haemacytometer for cell counting. The number of cells were counted under the inverted microscope (Optika DM-15, Italy).

#### Chlorophyll-A and phaeophytin determination

Chlorophyll-A and phaeophytin were analyzed by a spectrophotometric method. 10 ml of sample species was filtered with milipore size membrane filter paper attached to filter milipore titration units and connected to a vacuum pump. Dried filter extract was folded and placed in test tubes containing 15 ml of acetone 90% and was left to be degraded for up to 30 min. The samples were then transferred into cuvette to measure the absorbance using spectrophotometer at 664 nm wavelength. The cuvette was retrieved and 1–2 drops of hydrochloric acid (HCl) was added and the reading was taken once again using spectrophotometer on the same wavelength. The chlorophyll-A content and phaeophytin were determined according to Eqs. 3 and 4 as follows:

3 $${\text{Chlorophyll - A}}\,\left( {\text{mg/L}} \right) = \left( {{\text{A}}_{{\text{b}}} {-}{\text{A}}_{{\text{a}}} } \right) \times 2.43 \times 10.48 \times {\text{V}}/{\text{L}}$$

4 $${\text{Phaeophytin}}\,\left( {\text{mg/L}} \right) = \left[ {{\text{A}}_{{\text{b}}} {-}2.43\left( {{\text{A}}_{{\text{b}}} - {\text{A}}_{{\text{a}}} } \right)} \right] \times 10.48 \times 1.7 \times {\text{V}}$$

where A_b_ is the optical density readings before addition of HCl, A_a_ is the optical density readings after addition of HCl, V is the volume (ml) acetone (90% wt%, concentration) used (15 ml) and L is the width (cm) of cuvette (1 cm).

### Optimization statistical analysis

An optimization study using central composite design (CCD) was conducted with four operating parameters including gas flow rate, temperature, luminance and pH to obtain the maximum carbon fixation rate ability of *Isochrysis* sp. The level of each parameter is shown in Table 3. The response or results gained from experimental work were then analysed by Design Expert 7.0 (Stat Ease Inc. Minneapolis). The ANOVA analysis will be interpreted to understand the effects of each parameters towards the highest carbon fixation rate.

**Table 3:** Range of each operating parameters.

Operating parameters	Range of operating parameters				
	− 2	− 2	0	+ 1	+ 2
pH	4	5	6	7	8
Temperature (°C)	20	25	30	35	40
Gas flow rate (L/min)	0.05	0.10	0.15	0.20	0.25
Luminance (lux)	500	1000	1500	2000	2500

### CO_2_ fixation test under actual flue gas exposure

**Figure 4:**
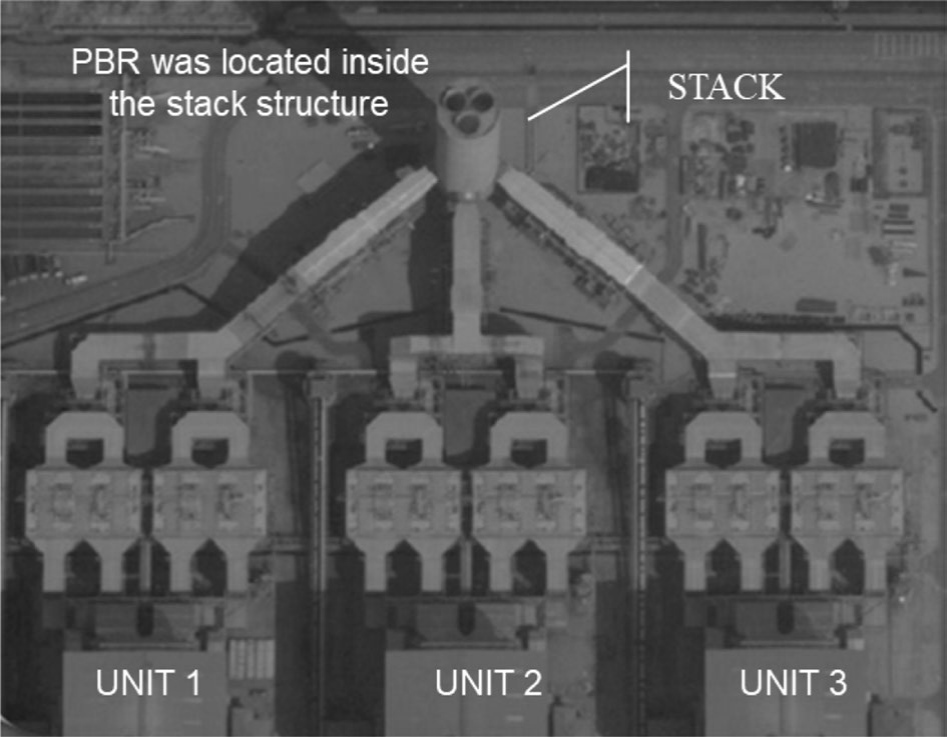
Location of the photobioreactor inside the stack structure.

A portion of flue gas generated at the power plant was tapped at the existing emission monitoring analyzer. The gas flow was maintained at 0.15 ± 0.03 L/min inside the culture. The experimental setup was placed inside the main stack which received the emissions from the three combustion unit as illustrated in Fig. 4. Each experiment was cultivated in 2 × 10L customized photo-bioreactor for maximum of 14 days. The culture was supplied with light for 12 h with an average luminosity of 15 µmol/m^−2^ s^−1^ at an ambient temperature range of ± 28 °C and a pH range of 6–7.

## Results and discussion

### Dominant microalgae species

The distribution of dominant microalgae species from the vicinity of a coal-fired power plant consisted of several different types of algae include diatom, cyanophyceae, blue-green algae, dinoflagellate and ciliophoran as tabulated in Table 4. The screening of dominant species is crucial to ensure the availability of algae in future use and its robustness to grow at the surrounding ambient.

**Table 4:** Microalgae distribution^a^, expressed as the mean percentage of community, chlorophyll a and phaeophytin values.

Type	Percentage (%)		
	Site 1	Site 2	Site 3
**A. Diatom**	19.4	18.1	13.6
1. Rhizosoleniaceae	8.4	9.3	2
2. Cheatoceraceae	6.2	5.1	2
3. Bacteriastraceae	0.1	1.2	1
4. Nitzschiaceae	0.1	0.1	1
5. Coscinodiscaceae	1.1	1.1	1
6. Naviculaceae	–	Tr	–
7. Surirellaceae	Tr	Tr	–
8. Thalassiosiraceae	–	Tr	0.5
9. Biddulphiaceae	0.2	0.3	0.7
10. Asterionellaceae	0.3	0.2	1.3
11. Dictyplaceae	0.4	0.5	1.3
12. Eucanpiaceae	0.4	0.3	0.4
13. Fragilariaceae	0.1	–	1.3
14. Hemialceae	0.6	–	–
15. Lauderiaceae	0.7	–	1.1
16. Pleurosigmaceae	0.1	–	–
17. Skeletonemaceae	0.4	–	–
18. Thallasionemaceae	0.3	–	Tr
**B. Cyanophyceae**	30	1	15
1. *Trichodesmium thiebautie*	30	1	15
**C. Blue-green algae**	42	56	60
1. *Nannochloropsis* sp.	1	5	5
2. *Tetraselmis* sp.	0.5	0.5	5
3. *Chlorella* sp.	Tr	Tr	–
4. *Isochrysis* sp.	40.5	50.5	50
**D. Dinoflagellate**	2.5	Tr	5.7
1. *Peridinium* sp.	0.5	Tr	3.1
2. *Ceratium* sp.	0.5	Tr	0.7
3. *Dinophysis* sp.	0.5	Tr	0.5
4. *Protoperidinium* sp.	0.5	Tr	0.7
5. *Gaunyaulax* sp.	0.5	Tr	0.7
**E. Ciliophora** sp.	4.2	5	4.1
1. *Thintinnopsis* sp.	2.8	4	1
2. *Favella* sp.	1.2	1	1
3. *Codonellopsis* sp.	0.2	Tr	1.1
4. *Epiplocylis* sp.	–	Tr	1
Total density (× 10^4^ cells/L )	5.4	6.8	64.3
Chlorophyll A (mg/m^3^)	0.2	0.3	0.61
Phaeophytin (mg/m^3^)	0.1	0.1	0.60

^a^Values are means of duplicate or triplicate analysed. Standard deviations are omitted for clarity, were normally < 5% (Tr—trace amount, less than 0.05%).

The population of Cyanophyceae and Blue-green algae were found to be dominated at all the selected sites, where the highest population of the species was identified at Site 3. Chlorophyll is a pigment that responsible for the photosynthesis process and phaeophytin is one of the breakdown products of chlorophyll. A high amount of these two elements indicates a higher population of microalgae^21,22^. The measurements of chlorophyll A and phaeophytin were at the highest in Site 3 which had the highest cell count of 64.3 × 10^4^ cells/L. This reading might be due to site 3, which is located at the Perak river’s mouth, which enriches nutrients from upstream discharges as indicated by the higher amount of phosphate content than the other two sites. Moreover, the identified Cyanophyceae and Blue-green algae species in all samples were *Trichodesmium thiebautie*, *Nannochloropsis* sp., *Tetraselmis* sp., *Isochrysis* sp., and traces amount of *Chlorella* sp. According to the microalgae population listed in Table 4, *Isochrysis* sp. was the dominant species within all the sample locations, which amounted up to 40–50% of the total population count. *Isochrysis* sp. belongs to the microalgae class of *Prymnesiophyceae* which is a flagellate cell-type with dominant golden brown pigment. It has a cell volume of 50–60 µm^3^ with an average diameter of 5–6 µm and a spherical rounded shape. Out of these blue-green algae species identified, three of them—*Nannochloropsis* sp., *Tetraselmis* sp. and *Isochrysis* sp. are commonly cited in various literature, discussing and highlighting their capability in producing good quality of biomass yield, lipid content, nutritional values and antioxidant properties^7,23,24^. Thus, these local species can be considered potential microalgal biomass for scale-up and further studies on the rate and optimization of CO_2_ fixation from a coal-fired power station in Malaysia.

### Screening of carbon fixation abilities

**Figure 5:**
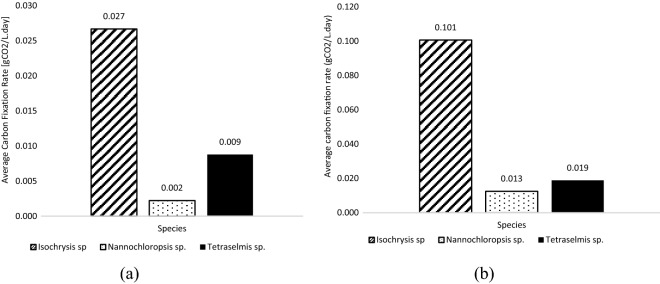
Carbon fixation rate of microalgae species in 2-L culture with (**a**) ambient air (**b**) pure CO_2_.

Three dominant isolated species, *Nannochloropsis* sp., *Tetraselmis* sp., and *Isochrysis* sp., were further scale-up and tested with ambient air and pure CO_2_ gas to screen for carbon fixation ability. *Isochrysis* sp. showed superior result in carbon fixation rate ability followed by *Tetraselmis* sp. and *Nannochloropsis* sp., as shown in Fig. 5a, b. This explained the dominancy of *Isochrysis* sp. in all the samples. The species is robust with the harsh condition of the power plant’s surroundings containing slightly higher CO_2_ concentration in its ambient.

It can be observed that *Isochrysis* sp. superseded *Nannochloropsis* sp. and *Tetraselmis* sp. in both culture environments; ambient air and pure CO_2_. The growth rate characteristic of these three species was also studied and their doubling time was determined using Eq. 4. Doubling time indicates the growth rate of a species and the rate of CO_2_ consumed. The results summarized in Table 5 strengthen the superiority of *Isochrysis* sp. as a better CO_2_ fixer where its doubling time is only about two days compared to *Nannochloropsis* sp. and *Tetraselmis* sp*.* that took up to five to seven days under pure CO_2_ exposure. The algae’s doubling time is affected by various parameters, such as temperature, pH, sunlight, and CO_2_ concentration. The shorter period of doubling time indicates that the species is fast-growing algae and can utilize higher CO_2_ as reflects its higher cell density^25–27^.

**Table 5:** Percentage of carbon fixation abilities of selected microalgae.

	*Isochrysis* sp.	*Tetraselmis* sp.	*Nannochloropsis* sp.
CO_2_ fixation rate (gCO_2_/L day)	0.101	0.019	0.013
Doubling time (days)	1.99	5.72	7.21

### Optimizing the *Isochrysis* sp. carbon fixation

Based on the screening of potential carbon fixation for the three species under ambient air and pure CO_2_ exposure, *Isochrysis* sp. was found to have the highest CO_2_ fixation rate*.* Thus, *Isochrysis* sp. was further optimized and exposed with simulated flue gas containing 4% CO_2_, 3% O_2_, 105 mg/m^3^ CO, and 272 mg/m^3^ NO_2_ at 2 × 10L lab-scaled photobioreactor. This approach was crucial for adaptation of the species before being tested with actual flue gas as higher CO_2_ concentration will not only improve the photosynthesis rate, however it could also lead to the acidification of the culture. Selection of suitable species that can tolerate with low pH and able to multiply within shorter doubling time is among the crucial parameters to ensure the survival of the culture under actual flue gas exposure as some of the research indicated that the algae culture was inhibited even with 5% of CO_2_ concentration^28,29^. In this study, 21 experimental runs were conducted as tabulated by Design Expert Software as in Table 6 to study the interaction effects of operating parameters on *Isochrysis* sp. CO_2_ fixation rate.

**Table 6:** Experimental results using interaction of operating parameters.

Run	Factor 1 (A) temperature (°C)	Factor 2 (B) pH	Factor 3 (C) gas flow rate (L/min)	Factor 4 (D) luminance (lux)	Response (Y) CO_2_ fixation rate (gCO_2_/L day)
1	30.00	6.00	0.15	2500.00	0.088
2	30.00	6.00	0.25	1500.00	0.031
3	30.00	6.00	0.15	1500.00	0.144
4	35.00	5.00	0.10	2000.00	0.000
5	20.00	6.00	0.15	1500.00	0.035
6	30.00	6.00	0.15	1500.00	0.151
7	35.00	7.00	0.10	1000.00	0.350
8	35.00	7.00	0.20	1000.00	0.321
9	30.00	8.00	0.15	1500.00	0.260
10	25.00	7.00	0.20	2000.00	0.249
11	30.00	6.00	0.15	1500.00	0.132
12	30.00	4.00	0.15	1500.00	0.000
13	30.00	6.00	0.15	1500.00	0.121
14	30.00	6.00	0.15	500.00	0.049
15	35.00	5.00	0.20	2000.00	0.037
16	25.00	5.00	0.10	1000.00	0.000
17	30.00	6.00	0.15	1500.00	0.125
18	40.00	6.00	0.15	1500.00	0.000
19	25.00	7.00	0.10	2000.00	0.347
20	25.00	5.00	0.20	1000.00	0.000
21	30.00	6.00	0.05	1500.00	0.125

The interaction with four parameters were analyzed by Response Surface Methodology (RSM) approach to determine the optimum parameters for the highest carbon fixation rate of *Isochrysis* sp. In predicting the optimal values of CO_2_ fixation rate within the experimental constrains, the experimental results were analyzed by regression analysis consisting of the effects of linear, quadratic and interaction which gave the following regression equation:

5 $$\begin{aligned} {\text{Y}} & = 0.82 + 0.037{\text{A}} + 0.12{\text{B}}{-}0.047{\text{C}} \\ & \quad {-}\left[ {1.00\left( {10^{ - 2} } \right)} \right]{\text{D}}{-}\left[ {3.75\left( {10^{ - 3} } \right)} \right]{\text{AB}}{-}0.031{\text{AC}} - \left[ {1.25\left( {10^{ - 3} } \right)} \right]{\text{AD}} \\ & \quad {-} \, 0.019{\text{BC}} + 0.056{\text{BD}} + 6.25\left( {10^{ - 3} } \right){\text{CD}}{-}0.088{\text{A}}^{2} {-}0.098{\text{B}}^{2} {-}0.16{\text{C}}^{2} {-}0.017{\text{D}}^{2} \\ \end{aligned}$$

where Y is the CO_2_ fixation rate and A, B, C and D are the temperature, pH, gas flowrate and lighting respectively. It was found that the highest carbon fixation rate of 0.350 gCO_2_/L day was achieved at temperature 35 °C, gas flow rate of 0.10 L/min, pH 7, and luminosity of 1000lux. Significance and adequacy of the model was analyzed through the analysis of variance (ANOVA). The summary of ANOVA representing the results of the quadratic response surface model fitting is shown in Table 7. The quadratic regression model was highly significant, as evident by the low probability value (P_model_ > F = 0.0051). Overall model’s (quadratic) F-value of 9.80 as per Table 7 implies the model is significant.

**Table 7:** ANOVA for response surface quadratic model with CO_2_ fixation rate as a response.

Source	Sum of square	Degree of freedom	Mean square	F-value	*P*_value_ > F
Model	1.10	14	0.078	9.80	0.0051
A	0.011	1	0.011	1.41	0.2802
B	0.11	1	0.11	13.83	0.0099
C	0.035	1	0.035	4.40	0.0807
D	8.00 (10^−4^)	1	8.000 (10^−4^)	0.10	0.7624
AB	5.625 (10^−5^)	1	5.625 (10^−5^)	7.041 (10^−3^)	0.9359
AC	7.813 (10^−3^)	1	7.813 (10^−3^)	0.98	0.3609
AD	6.250 (10^−6^)	1	6.250 (10^−6^)	7.824 (10^−4^)	0.9786
BC	2.812 (10^−3^)	1	2.812 (10^−3^)	0.35	0.5746
BD	0.013	1	0.013	1.58	0.2549
CD	3.125 (10^−4^)	1	3.125 (10^−4^)	0.039	0.8497
A^2^	0.20	1	0.20	24.45	0.0026
B^2^	0.24	1	0.24	30.31	0.0015
C^2^	0.62	1	0.62	77.41	0.0001
D^2^	7.227 (10^−3^)	1	7.227 (10^−3^)	0.90	0.3783
Residual	0.048	6	7.989 (10^−3^)	–	–
Lack of fit	0.047	2	0.024	182.36	0.0001
Pure error	5.200 (10^−4^)	4	1.300 (10^−4^)	–	–
Corrected total	1.14	20	–	–	–

In favor to the optimization of CO_2_ fixation rate, B, A^2^, B^2^ and C^2^ were the significant model terms, which indicates that these parameters have a significant contribution towards achieving the highest CO_2_ fixation rate. At the model level, the correlation measure for estimating the regression equation is the determination of coefficient, *R*^2^. The coefficient of *R*^2^ determines the goodness of the model fitting. In this study, the value of *R*^2^ is 0.9581 as shown in Table 8, indicates a better correlation between observed and predicted values where only 4.19% of variations were not explained by the model. The coefficient of variation (CV) indicates the degree of precision with which the treatments are compared. Usually, the higher the value of the CV, the lower is the reliability of the experiment. In this study, the value of CV was 16.51%, which indicated a small residue between actual and predicted values of CO_2_ fixation rate. The adequate precision value for this study is 9.553, which measured the signal to noise ratio. A ratio greater than 4 is desirable as it gives better precision and reliability of the carried out experiments^30^.

**Table 8:** Analysis of model fitting.

Elements	Values
Standard deviation (SD)	0.089
Mean	0.54
C.V. %	16.51
PRESS	1.97
R^2^ (R-squared)	0.9581
Adjusted R^2^ (adj R-squared)	0.8603
Predicted R^2^ (pred R-squared)	− 0.7214
Adeq precision	9.553

**Figure 6:**
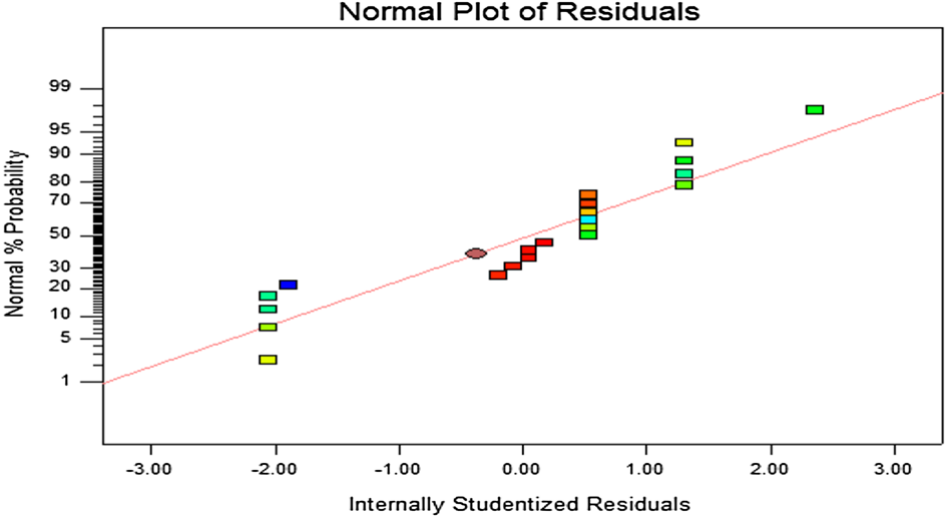
Normal probability plot for the residuals from CO_2_ fixation rate model.

The normal probability plot of residuals and the plot of residuals versus predicted values of the response for the CO_2_ fixation rate are shown in Fig. 6. A satisfactory correlation between actual and predictive values was presented, as distribution of plots was balanced throughout the linear line, indicating a good fit of the model.

**Figure 7:**
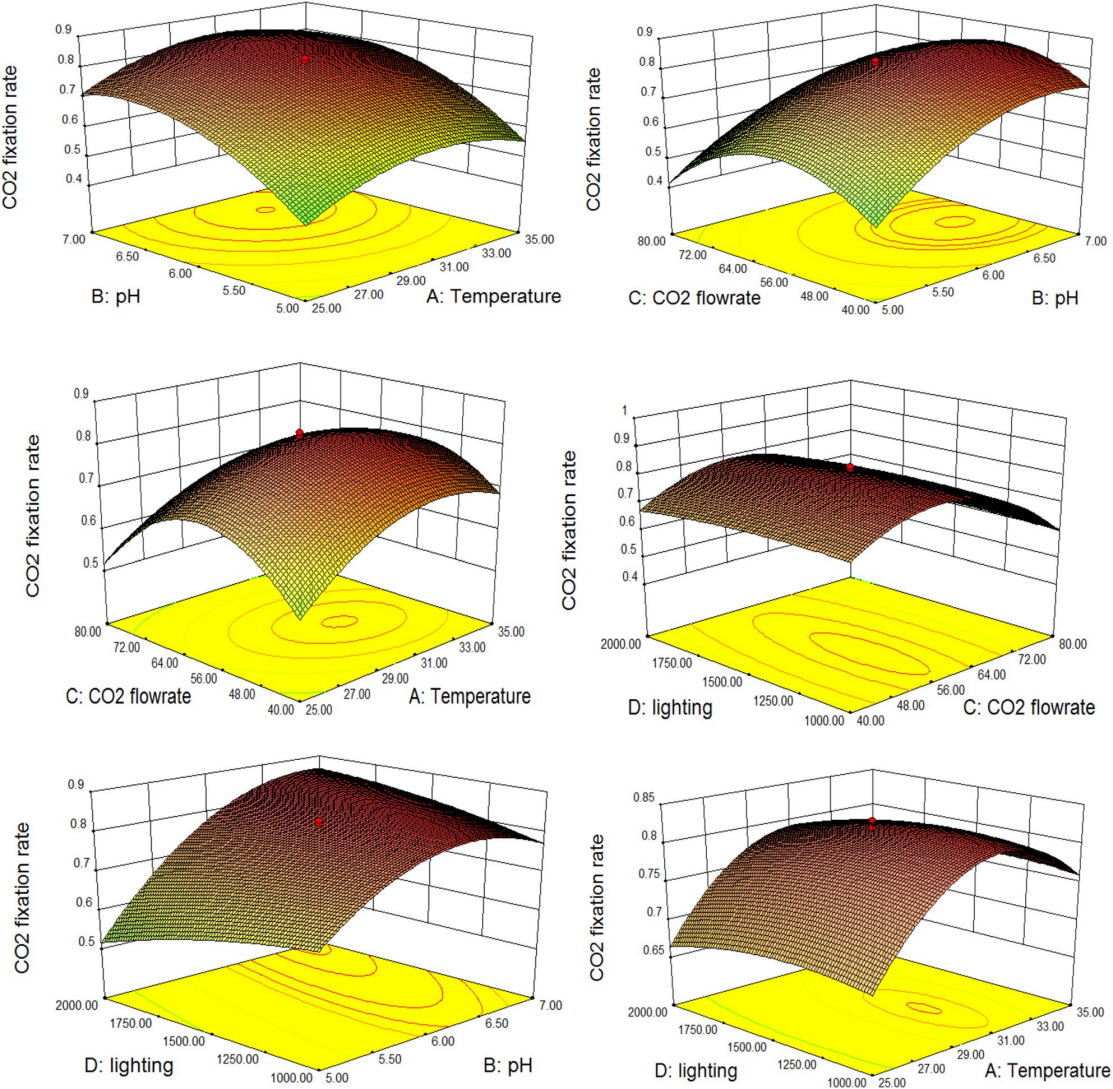
Optimum interaction of operating parameters towards highest carbon fixation rate.

The 3D response surface and 2D contour plots are graphical representation of the regression equation to determine the optimum values of the variables^31–33^. Interaction of each operating parameters in achieving highest carbon fixation rate of *Isochrysis* sp. is presented in 2D contour plots and 3D response surface as in Fig. 7. The maximum activity was obtained near the center points of response surface.

According to Fig. 7, the predicted highest CO_2_ fixation rate was at pH 7.5, temperature 30 °C, luminance of 1500lux and 0.15L/min gas flow rate. A validation experiment was carried out to verify these optimum parameters and the ability of *Isochrysis* sp. carbon fixation rate was further improved by 6% with the carbon fixation rate of 0.370 gCO_2_/L day.

### Effects of operating parameters

Based on 2D contour plots and 3D response surface as in Fig. 7, the optimal temperature to enhance *Isochrysis* sp. growth is at 30 °C. The rate of microalgae growth was retarded as the temperature decreases and tend to inhibit as the temperature rises. The range of optimal temperature varies depending on the species, however, most of the microalgae species have an optimum temperature in a range of 20–30 °C^20^. Determination of optimal temperature is crucial to ensure the survival of selected microalgae during outdoor cultivation as it will be exposed to a large fluctuation in temperature and excessive heat will create shear stress that can disrupt microalgae cell wall^34,35^. The optimum range for *Isochrysis* sp. was at pH 7–8^36,37^. This also agreed well with the observation from Table 7 that pH has a significant influence on the CO_2_ fixation rate as indicated by the values of Prob > F, which was less than 0.0500. The CO_2_ and SO_2_ solubility highly contributes to the variation of pH value as the growth will be affected by the culture’s acidity due to simulated flue gas exposure. This acidic environment may retard and inhibit the growth of microalgae^38,39^. It is important to control the gas flow rate to moderate the effect of acidic environment in the culture. In this study, the optimum flue gas flow rate was achieved at 0.15 L/min. Higher gas flow rate contributes to the decrement of pH value and produces hydrodynamic stress to the algae, which will inhibit the culture. An optimum gas flow rate is also crucial in maintaining the homogeneity of the culture. Compared to other operating parameters, illuminance gave less impact to microalgae culture in this study as the experiment was conducted indoor and the gap between readings are quite small. Based on studies conducted at the outdoor condition under direct sunlight, the optimum range of luminosity is in a range between 5000 and 10,000 klux^20^.

### Carbon fixation ability under actual coal-fired flue gas exposure

**Figure 8:**
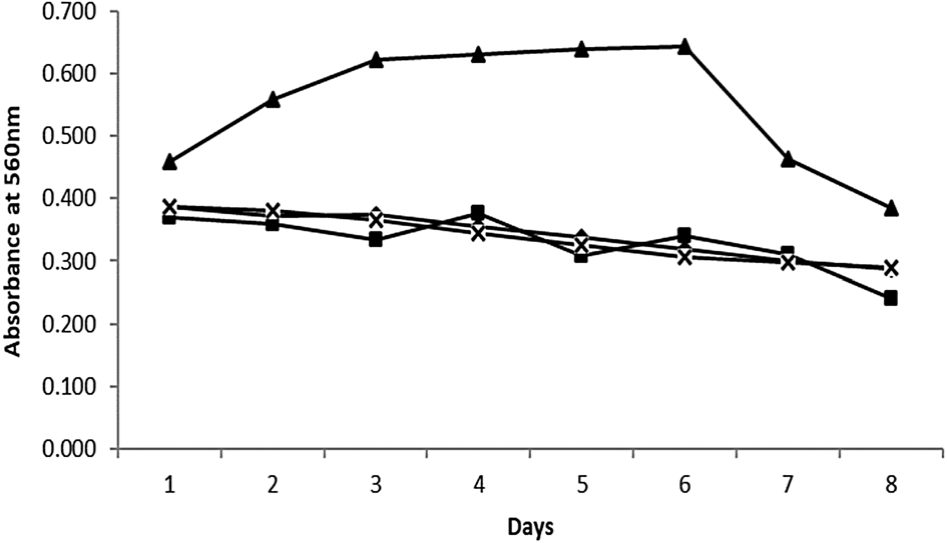
Growth rate characteristic of *Isochrysis* sp. under actual flue gas condition. Culture condition: (multi sign) control (filled diamond) first culture: initial OD 0.387, (filled square) second culture: initial OD 0.369, (filled triangle) third culture: initial OD 0.459.

*Isochrysis* sp. had shown its capability as a carbon fixer under ambient air and simulated flue gas exposure; thus, it was further tested under the power plant’s actual flue gas. The cultures were subjected to cycles of growth phase to observe the growth adaptability of *Isochrysis* sp. under harsh flue gas condition containing on average of 4.08% O_2_, 200.21 mg/m^3^ SO_2_, 212.29 mg/m^3^ NO_2_, 4.73% CO_2_ and 50.72 mg/m^3^ CO throughout the culture period. Figure 8 shows the four batches of *Isochrysis* sp. culture using a 2 × 10L customized photobioreactor skid and each cycle lasted up to 8 days. The control culture was first acclimatized using aeration before being exposed to actual flue gas. The control culture and the first two batches under flue gas exposure showed a stagnant growth, which indicates a gradual adaptation of the cultures with the elevated CO_2_ concentration in the actual coal-fired flue gas, as shown in Fig. 8. This adaptation might also happen due to the flue gas pollutants such as SO_2_, NO_2_, and particulate matter. Some studies indicated that these pollutants could inhibit microalgae growth due to decrement in pH value when SO_2_ hydrolysis happens^40–42^. On the other hand, at certain concentrations, NO_2_ and particulate matter can be transformed into nutrient and minerals sources for microalgae and promote its growth^43,44^. However, different species showed different effects on these pollutants, as some studies demonstrated no significant effects on microalgae growth^45^. However, the microalgae growth in this study was not influenced by the pollutant concentrations due to their intrinsic characteristics.

**Figure 9:**
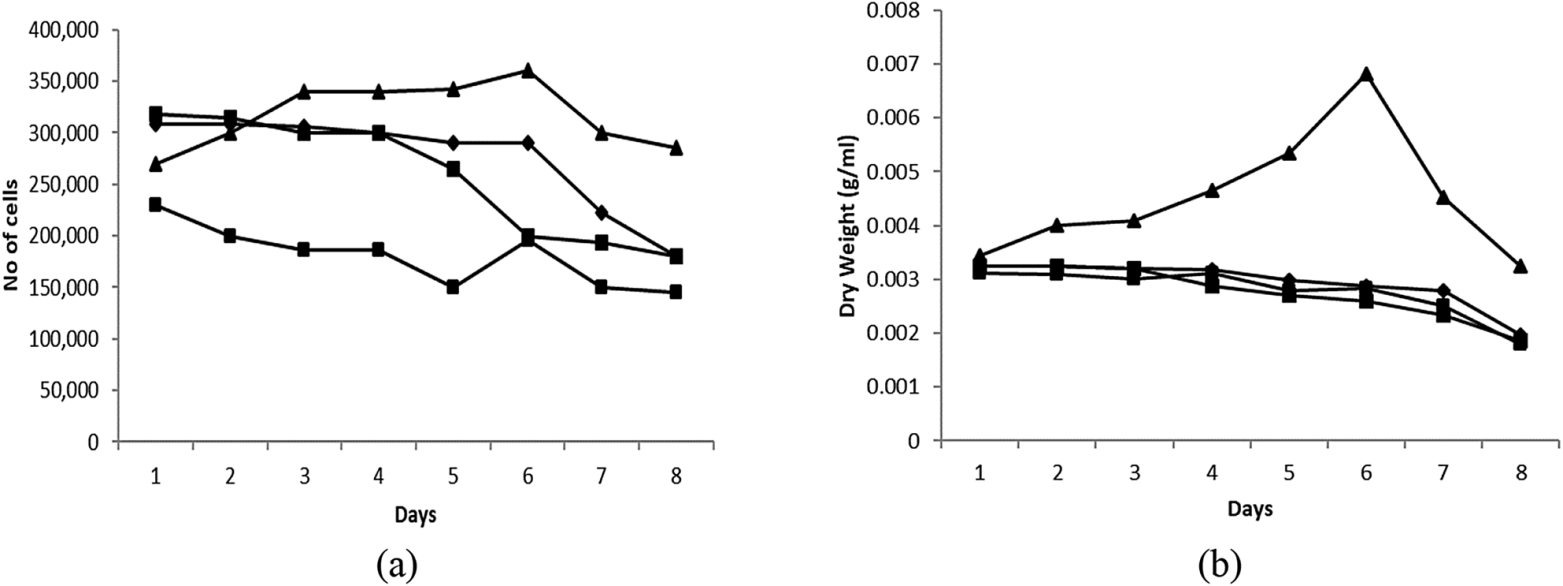
(**a**) No of cell profile (**b**) Dry weight profile for *Isochrysis* sp. under actual flue gas condition Culture condition: (multi sign) control (filled diamond) first culture: initial OD 0.387, (filled square) second culture: initial OD 0.369, (filled triangle) third culture: initial OD 0.459.

As shown in Fig. 8, after almost 3 weeks of acclimatization phase, the third culture with a slightly higher initial culture density demonstrated the survival of the species under the influence of harsh flue gas conditions. This can be observed by the appearance of the log phase with an increment in optical density, number of cells, and dry weight of the culture as shown in Figs. 8 and 9. This was also supported by a few research that suggested the gradual adaptation of microalgae over high CO_2_ concentration. A study carried out by Aslam et al. (2017) took 2 to 4 weeks adaptation period before the mixed freshwater dominated by *Desmodesmus* sp. was tested under actual flue gas containing 11.24% CO_2_. The maximum rate of *Isochrysis* sp. carbon fixation was achieved at 0.35 gCO_2_/L day under this actual flue gas exposure.

**Figure 10:**
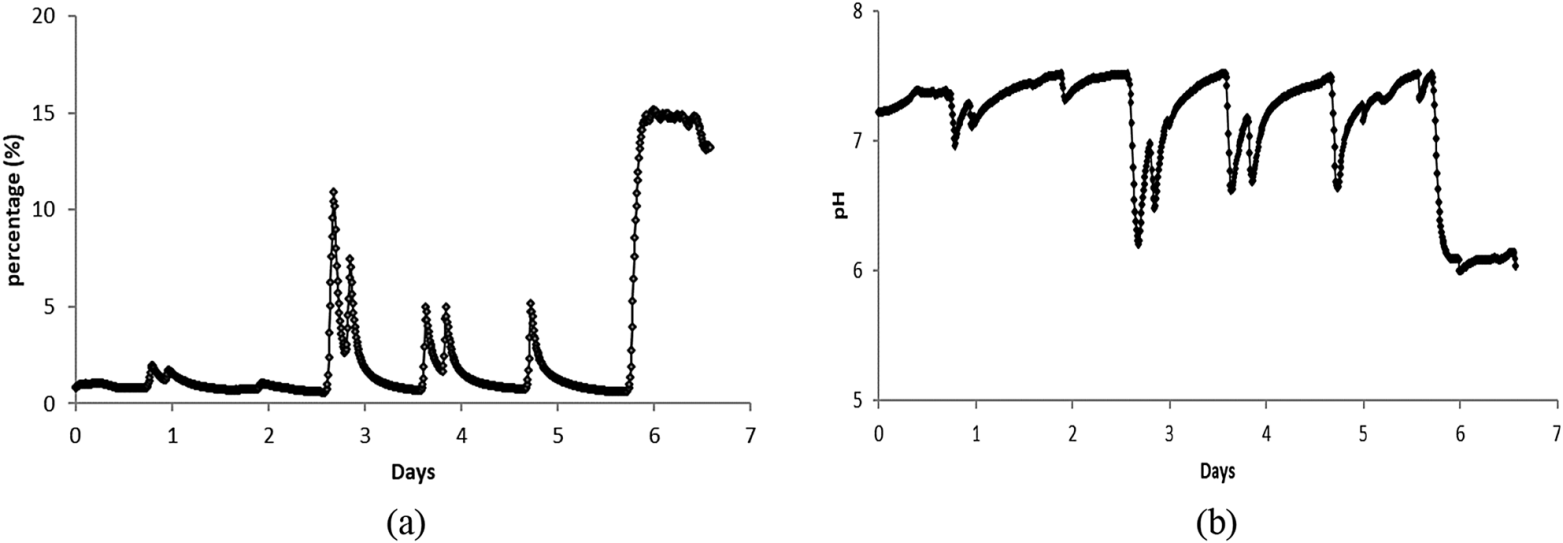
(**a**) Dissolved CO_2_ profile (**b**) pH profile.

However, for the last two days of the culture period, the growth of culture was retarded and started to enter a decay phase. This phenomenon happened due to the decrement of pH value, below pH 6 in the culture. As observed in Fig. 10, dissolved CO_2_ was rapidly increased in the last two days of the culture up to 15.2% and resulting in the pH dropped which further inhibit the culture growth. This was also supported by the declination of cell density and cell count by about 40%.

Table 9 summaries several studies reported by other workers using similar dominant species in this study. These data illustrated that higher biomass productivity and CO_2_ fixation rate can be achieved with higher CO_2_ concentration. The results from this study can be considered quite low as it is a preliminary effort to investigate the potential of indigenous species for CO_2_ mitigation. There are few strategies and parameters to address in improving the microalgae productivity such as enhancing culture condition, using consortium microalgae species and improving photobioreactor design^43,44,46^. The improvement of microalgae productivity will further enhance the CO_2_ fixation ability.

**Table 9:** Comparison of CO_2_ fixation ability of microalgae species reported in the literature.

Microalgae species	CO_2_ (%)	Biomass productivity (g L^−1^ d^−1^)	CO_2_ fixation rate (gCO_2_ L^−1^ d^−1^)	Volume culture (L)	Reference
*Tetraselmis suecica*	N.D	0.46	0.8657^a^	120	^40^
*Tetraselmis* sp.	N.D	0.42	0.7904^a^	1000	^41^
*Nannochloropsis* sp.	15	0.27	0.5081^a^	N.D	^42^
*Nannochloropsis* sp.	2–15	0.49	0.9222^a^	N.D	^43^
*Isochrysis* sp.	N.D	0.31	0.5834^a^	120	^40^
*Isochrysis galbana* (T-iso)	N.D	0.113	0.2127^a^	50	^44^
*Isochrysis galbana* ALII-4	N.D	0.32	0.6022^a^	N.D	^45^
*Isochrysis* sp.	4.73	0.19	0.3500	10	This study

^a^Calculated from the biomass productivity according to the following equation: CO_2_ fixation rate (Pco_2_) = 1.88 × biomass productivity (mg L − 1 d − 1)^46^.

## Conclusions

The findings from this study demonstrated proof of concept on the application of microalgae as the biological agent for carbon fixation towards sustainable coal-fired power generation by reducing the CO_2_ emission. In this study, dominant indigenous species from the vicinity of Malaysian coal-fired power plant were screened and tested in the laboratory for their fixation capabilities. The interaction of four operating parameters was analyzed by Response Surface Methodology (RSM) approach to determine the highest carbon fixation rate of *Isochrysis* sp. This superior microalgae species was then adapted to cycles of growth phase under harsh flue gas exposure from coal combustion at Sultan Azlan Shah Power Station, Manjung, Perak. *Isochrysis* sp. had shown its capability as a carbon fixer under actual flue gas exposure after a certain period of acclimatization. The downstream application of algae biomass in producing valuable downstream products could also be explored to promote industrial symbiosis. With several improvements, including culture techniques, photobioreactor design, and scale-up parameters, microalgae could become a sustainable solution in neutralizing carbon emission from power plants in the years to come.
